# Treatment Preferences in Acute Allergic Reactions—How Can the Context Impact the Preference for a Corticosteroid Mouth Film Versus Conventional Tablets?

**DOI:** 10.3390/jmahp14030049

**Published:** 2026-08-11

**Authors:** Andrea Karadak, Karin Wahlberg, Göran Tornling, James Kereki, Leif Bjermer, Jonas Hjelmgren

**Affiliations:** 1 The Swedish Institute for Health Economics, SE-223 61 Lund, Sweden; andrea.karadak@ihe.se (A.K.);; 2 Division of Immunology and Respiratory Medicine, Department of Medicine Solna, Karolinska Institutet, SE-171 76 Stockholm, Sweden; 3 AcuCort AB, SE-223 81 Lund, Sweden; 4 Department of Respiratory Medicine and Allergology, Lund University, SE-221 85 Lund, Sweden

**Keywords:** acute allergic reactions, corticosteroids, preferences, ex ante, interim, ex post

## Abstract

Background: The objective was to assess how preferences for a self-dissolving dexamethasone mouth film versus conventional tablets for acute allergic reactions may vary according to population characteristics and the level of outcome knowledge for the intervention (i.e., ex ante, interim, and ex post). Methods: We used preference data from two studies: a questionnaire study with a choice between mouth film and tablets based on a description of the mouth film (ex ante), and a clinical trial with ratings of accessibility and perceived security when the mouth film was available (interim) or used for acute allergic reactions (ex post). Associations between preferences and participant characteristics (demographics and clinical history) were analyzed using logistic regression models. Results: Preference for the mouth film over tablets remained strong as outcome information increased from ex ante to ex post and was consistent across study populations. Higher relative preference was associated with female sex, longer disease duration, older age, and prior severe reactions, whereas satisfaction with current treatment and regular allergy check-ups were linked to weaker relative preference. Conclusions: Preference for the mouth film over tablets was stable, but the degree of preference may depend on characteristics that influence sensitivity to treatment challenges in acute situations.

## 1. Introduction

Certain allergens, such as foods, insect venoms, animal fur and pollen, can trigger acute allergic reactions (AARs) [1]. The severity of AARs can vary from mild itching of the eyes, nose, mouth, and throat to the potentially life-threatening anaphylaxis (i.e., anaphylactic shock) with symptoms in the airways and the cardiovascular system [2,3].

Timely treatment is important to prevent the reaction from progressing in severity. Notably, the mortality rate from anaphylaxis has been reported to be around 0.5 per million person-years [4], and the fatality rate among hospitalizations or emergency department presentations for anaphylaxis in the US has been estimated to be around 0.3% [5]. Systemic corticosteroids (betamethasone, dexamethasone, or prednisolone) are part of the mainstay treatment for moderate-to-severe AARs [6]. In the most severe reactions, corticosteroids together with antihistamines are often used as adjunctive treatments after administration of epinephrine [3,7]. Corticosteroids are typically prescribed as tablets that require dissolution in water prior to oral administration. A typical dose is 5–15 betamethasone tablets (0.5 g) [8].

A new dexamethasone mouth film may provide a more accessible and faster treatment alternative to conventional corticosteroid tablets in AARs. The small mouth film fits in a mobile case and melts on the tongue without the need for water [9]. Two recently published Swedish studies, one questionnaire study [8,10] and one clinical trial [11], report a clear preference for the mouth film over tablets among individuals with experience of moderate-to-severe AARs and prior prescriptions for systemic corticosteroids.

In the questionnaire study, the preferences for the mouth film versus tablets were evaluated based on a hypothetical situation; the mouth film was described to the respondents but not provided. Thus, the study assessed the respondents’ expectations of the mouth film rather than actual experiences, which in health economic terms can be referred to as ex ante preferences (i.e., preferences before the outcome is known) [12].

In the clinical trial, treatment satisfaction and perceived security of the mouth film compared with tablets was assessed among participants who were provided with both the mouth film and tablets, which they could choose to use in case of an AAR. Most of the respondents did not experience an AAR over the trial period; these participants experienced having the mouth film available without using it, and their responses thus reflect interim preferences (i.e., based on partial outcome). In contrast, the smaller group of participants who experienced one or more ARRs over the trial period, which they treated with the mouth film, provide ex post preferences (i.e., based on full knowledge of the outcome).

In addition to providing information on treatment preferences for the mouth film versus tablets, the different circumstances provided in the studies allow us to examine if preferences may vary depending on the context. Moreover, the preferences may also reflect population characteristics, which could differ between studies depending on inclusion criteria and recruitment methods.

Preference studies are particularly relevant for treatments of AARs, where successful treatment depends not only on pharmacological effectiveness but also on patients’ willingness and ability to carry and administer the medication promptly during an emergency. Evaluating patient preferences for alternative administration forms, such as a mouth film compared with conventional tablets, may therefore provide insights into treatment attributes that are likely to influence real-world treatment use and support more patient-centered healthcare decisions.

In this study, we further explore data on treatment preferences for the mouth film versus tablets from the questionnaire study and the clinical trial to:

1. Identify potential factors related to demographics and clinical history that influence treatment preferences (i.e., mouth film versus tablets) among people with experience of AARs.
2. Assess whether the treatment preferences differ depending on the level of outcome information available (i.e., the difference between ex ante, interim and ex post preferences).

## 2. Methods

### 2.1. Study Design

#### 2.1.1. Overall Design

**Figure 1:**
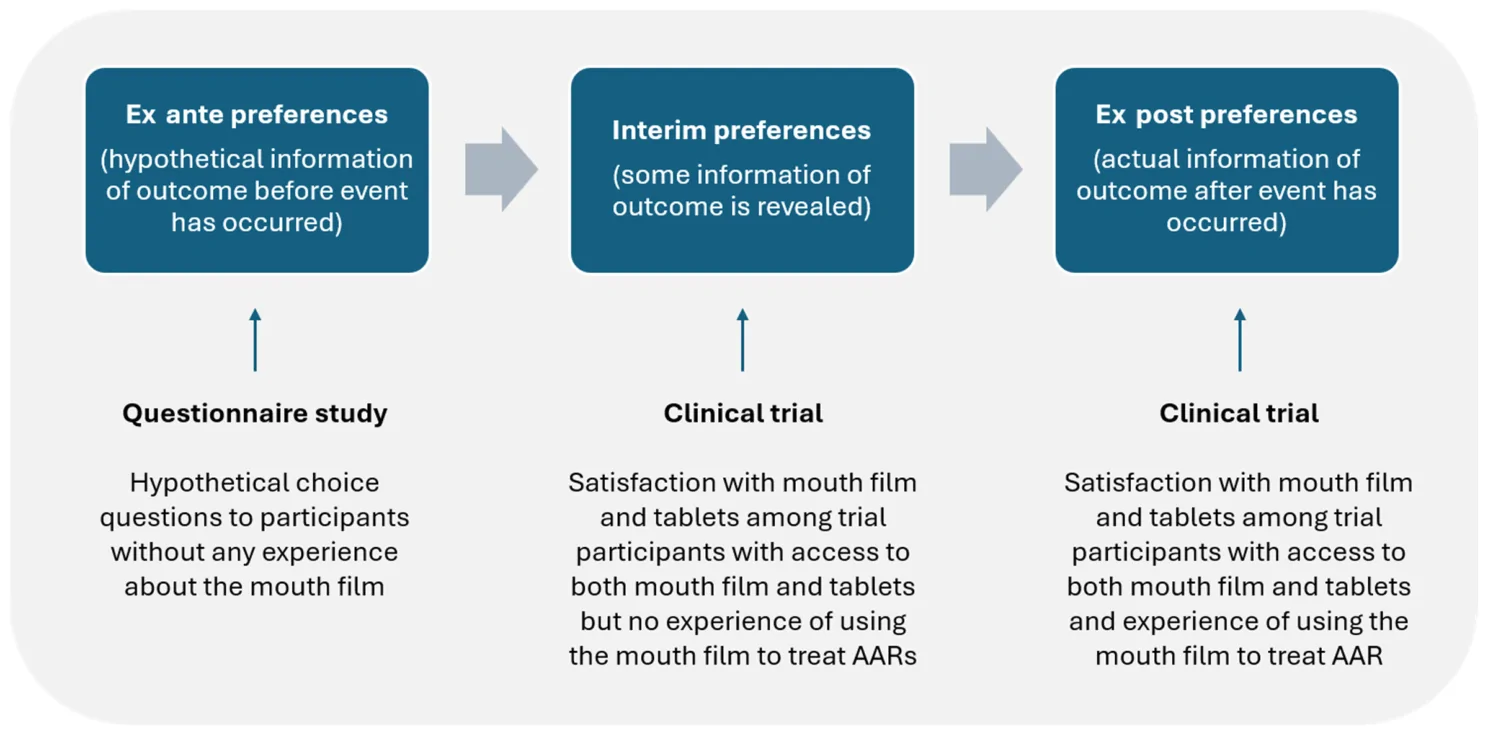
Illustration of how the studies included in the analyses represent different types of preferences. Abbreviation: AAR: acute allergic reaction.

The study included data from a questionnaire study and a clinical trial, which were used to represent different levels of treatment preferences according to established definitions as illustrated in Figure 1.

#### 2.1.2. Questionnaire Study

The questionnaire data was published in two parts focusing on disease burden and unmet needs [8], and treatment preferences by discrete choice experiment [10], respectively. The study was cross-sectional and used a 2-part web-based method for collection of data. The study population included adults (≥18 years) with self-reported allergic problems who had been prescribed corticosteroid tablets for AARs. Individuals reporting only allergy to pollen were excluded from the dataset based on the assumption that these individuals used corticosteroids for preventive treatment of allergic symptoms and not for acute treatment of moderate-to-severe AARs.

The main outcome from the questionnaire study used in the current analyses was preference for the mouth film versus tablets expressed as treatment choice based on the following question: If the effect and price were the same for mouth film and tablets, which alternative would you choose? Answer alternatives: mouth film/tablets.

#### 2.1.3. Clinical Trial

The clinical trial was a Phase IV, non-randomized, open-label, single-center, low-interventional trial conducted over six months at Kåbohälsan in Uppsala, Sweden (trial registration: EU CT no. 2023-504975-25-00). It included adults (≥18 years) with moderate-to-severe allergies currently receiving betamethasone tablets to be used in case of an AAR. Individuals reporting only allergy to pollen were excluded (see previous section). Participants had access to the investigational product (dexamethasone mouth film) and their standard treatment (betamethasone tablets) to use as rescue medication in case of an AAR. Instructions for how to use the mouth film were provided at the start of the trial.

The inclusion of relevant control variables was based on results from the questionnaire study, where variables that had a significant impact on preferences had priority over variables that were less sensitive [10]. Age and sex were additionally included given their potential association with variation in medication-carrying behavior, whereas prescribed epinephrine autoinjector use was included as a proxy indicator of elevated risk for more severe AARs. A more detailed description of the trial setup can be found in Bjermer et al. 2026 [11].

Participants’ characteristics were collected at baseline, and their experiences with the mouth film and tablets were recorded monthly in an electronic diary (eDiary). Thus, a participant who filled in the eDiary at every follow-up had a maximum of 6 observations.

Outcomes from the clinical trial used in the current study included preferences for the mouth film versus tablets expressed as satisfaction with accessibility and perceived security. These two outcomes are described in more detail below.

### 2.2. Preference Assessment and Statistical Analyses

#### 2.2.1. Questionnaire Study—Ex Ante Preferences

A logistic regression model was applied to evaluate variables influencing preference (based on choice) as a binary dependent outcome, where the mouth film was assigned a value of 1 and tablets a value of 0.

In a basic model, we included the independent variables that matched those available from the clinical trial, including participant characteristics (i.e., demographics, last AAR event, allergy duration, type of allergy, and clinical history), and concomitant prescription for an epinephrine autoinjector. The following variables were only available from the questionnaire study and were evaluated in an extended model: disease/treatment burden (i.e., swallowing problems, anxiety, fearing for your life from an AAR, regular healthcare check-ups and emergency visit(s) due to AAR) and experience of AARs and treatment (i.e., satisfied with current treatment, medication compliance, severity of AAR and number of tablets used for treatment of an AAR). More detailed variable descriptions can be found in the Supplementary Materials (Table S1A).

#### 2.2.2. Clinical Trial—Interim and Ex Post Preferences

We used an ordinal logistic regression model to analyze preferences for mouth film versus tablets expressed as satisfaction with access and perceived security. Since we were dealing with panel-like data where one single patient occurs up to six times (month 1–6), a clustered model accounting for variance within individuals was used.

Separate analyses were conducted for the two dependent variables reflecting different aspects of preference:

1. Satisfaction with access to treatment with mouth film and tablets, respectively, based on two questions: “How satisfied are you with how accessible your medication (the mouth film/the tablets) is in the event of an acute allergic reaction?” Answers were rated as follows: not at all satisfied = 0, a little = 1, neither nor = 2, satisfied = 4, and very satisfied = 4. One preference outcome variable was constructed by taking the difference between the rating for the mouth film and the corresponding rating for the tablets for each participant over the study period (months 1 to 6). This resulted in an ordinal scale ranging from −4 (tablets much better) to 4 (mouth film much better). A value of “0” indicates “indifference” between the mouth film and the tablets.
2. Perceived security with the mouth film versus tablets based on the question: “Do you feel safer when you have access to the mouth film as compared to tablets?” Answers were rated as follows: not at all safe = −2, less safe = −1, neither nor = 0, more safe = 1, and much more safe = 2.

The independent variables included in the base case models were participant characteristics (i.e., demographics, obesity, type of allergy, and clinical history) and concomitant prescription for an epinephrine autoinjector. More detailed variable descriptions can be found in the Supplementary Materials (Table S1B).

### 2.3. Model Selection

Model selection was based on a set of criteria. Log-likelihood values were compared across models, and the specification with the highest log-likelihood value was selected as the preferred model. Variable significance was assessed by examining the *p*-value of the included predictors, with variables considered statistically significant if their *p*-values were below 0.05. Predictors that were non-significant and did not influence the model specification were excluded. Multicollinearity was assessed using the Variance Inflation Factor (VIF). All VIF values were below conventional thresholds, indicating no serious multicollinearity. Correlation matrices were also examined for high pairwise correlations, which did not exceed conventional thresholds.

### 2.4. Additional Analysis

#### 2.4.1. Stability of Preferences over Time

A linear mixed-effects model was used to evaluate differences in accessibility ratings between the mouth film and the tablet formulation over the six-month follow-up period. Treatment formulation (the mouth film versus tablets), months (continuous), and the treatment-by-time interaction were included as fixed effects to assess both overall differences between formulations and potential changes in ratings over time. To account for repeated measurements within participants, the model included a participant-specific random intercept.

The analyses were performed on both the original dataset (344 observed, non-imputed observations) and a balanced dataset (492 observations after imputation). To obtain the balanced dataset for the linear mixed-effects model, missing observations within each participant were imputed using the Last Observation Carried Forward (LOCF) method. Because LOCF cannot be applied to leading missing values (i.e., those occurring before the first recorded observation), these values were instead imputed using the Next Observation Carried Backward (NOCB) method, which carries the first available measurement backward to earlier missing positions within each participant. This resulted in a balanced dataset comprising 492 observations (41 patients over 6 months with 2 treatment formulations).

#### 2.4.2. Interim Versus Ex Post Analyses

In a subgroup analysis with those individuals who experienced an AAR event and treated it with mouth film (i.e., ex post subgroup), statistical differences in ratings of satisfaction with accessibility and perceived security with mouth film versus tablets were analyzed between observations before the experience of an AAR event and after an AAR event (including the month of the AAR event). The same analysis was performed between observations in months in which an AAR event occurred and months in which no AAR event occurred. In the analyses, mean differences were evaluated using a paired two-tailed t-test, with *p*-values reported.

To get a balanced dataset for interim versus ex post preferences, missing observations were imputed by LOCF or NOCB as described in the section above. This generated a total of 60 observations (10 patients over 6 months).

## 3. Results

### 3.1. Participant Characteristics

Table 1 provides an overview of participant characteristics for the questionnaire study and the clinical trial. For the clinical trial, the numbers are presented for the whole study population and for two subgroups: one subgroup (ex post subgroup) included 10 participants who experienced one or more AARs during the trial, which they choose to treat with the mouth film, and one group (interim subgroup) with the remaining participants, including two participants who experienced one AAR each during the trial, which they chose to treat with tablets. Some information differed between studies: the questionnaire study contained less granular data on allergy duration and no data on BMI (or obesity).

Although not tested statistically, the data indicated some differences in participant characteristics between the questionnaire study and the clinical trial (Table 1). Most notably, the proportions with epinephrine autoinjector prescriptions were larger in the clinical trial compared with the questionnaire study, whereas proportions reporting long allergy duration (>5 years) and multiple types of allergies were larger in the questionnaire study. The two studies also differed in terms of types of allergies; pollen, animal, and food allergies were more common in the questionnaire study. Statistically significant differences between the interim and ex post subgroups in the clinical trial were represented by a higher mean BMI and obesity rate in the ex post group.

### 3.2. Treatment Preferences

#### Ex Ante Preferences—Analyses of Questionnaire Study Data

From the descriptive responses from the questionnaire study, 285 participants (74%) reported that they would choose the mouth film over the tablets, all else being equal. The remaining 102 participants (26%) reported that they would choose the tablets.

In the basic logistic regression model, a preference for the mouth film was significantly positively associated with longer disease duration and food allergies. Participants who reported allergy duration of more than 5 years were more likely to choose the mouth film over tablets compared to those with shorter allergy duration (*p* < 0.001). Similarly, participants with food-related allergies were more likely to choose the mouth film over tablets compared to those without food allergies (*p* = 0.004). The detailed regression matrix for the basic logistic regression model can be found in the Supplementary Materials (Table S2).

In addition to the associations with disease duration and food allergies observed in the basic model, the extended model also showed significant positive associations between a preference for the mouth film over tablets and experience of more severe AARs (*p* = 0.021), whereas being satisfied with current treatment (i.e., betamethasone tablets) (*p* = 0.014) and having regular health check-up (*p* < 0.001) was negatively associated with a preference for the mouth film. This means that participants who scored the severity of their most recent AAR in the higher range were more likely to choose the mouth film over tablets compared with those scoring the severity in the lower range. In contrast, participants who reported a higher degree of satisfaction with their current treatment were less likely to choose the mouth film over tablets compared with those who were less satisfied with their treatment. Similarly, participants reporting that they had regular health check-ups were also less likely to choose the mouth film compared to those not having regular check-ups. The detailed regression matrix for the ordinal logistic regression model can be found in the Supplementary Materials (Table S2).

**Figure 2:**
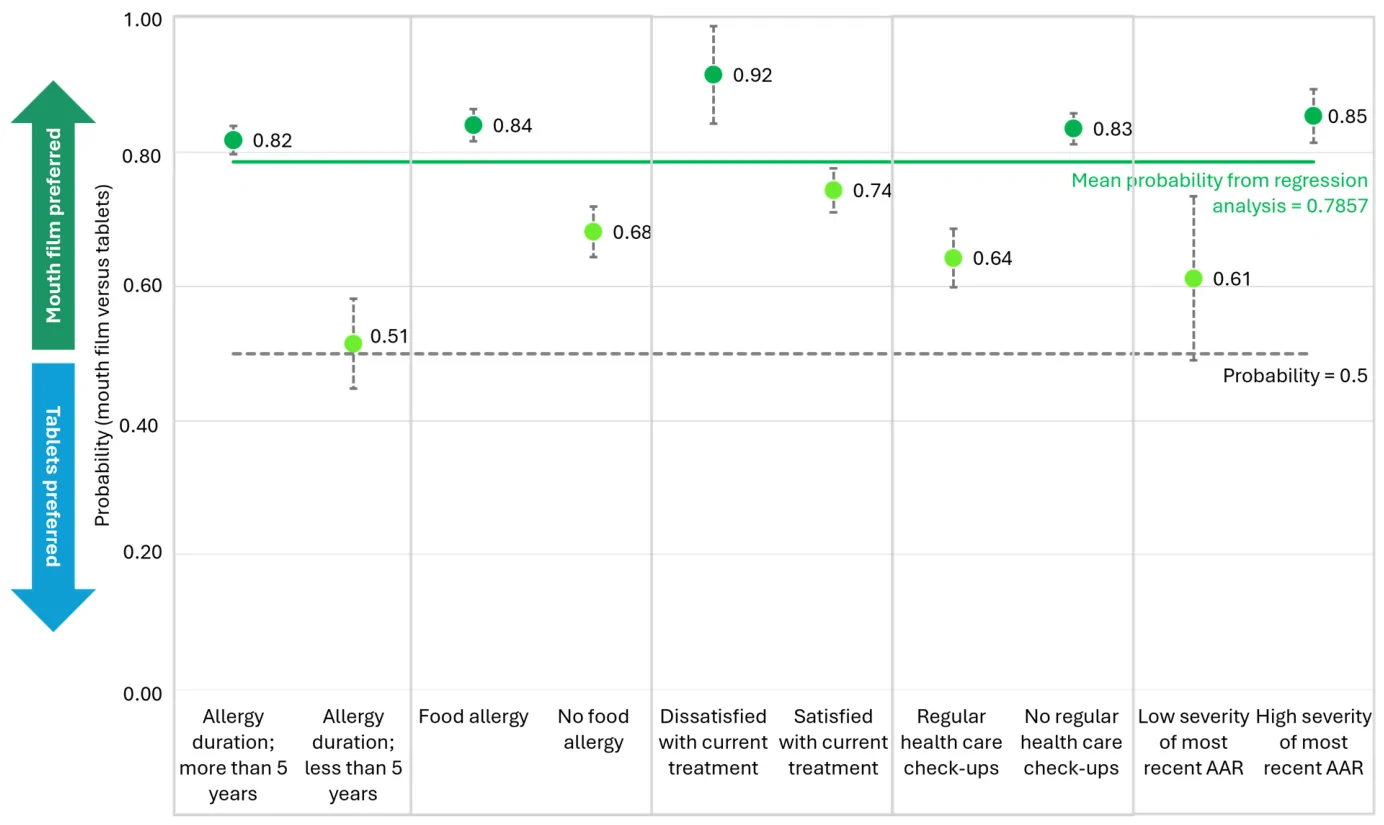
Probabilities for treatment choice of mouth film versus tablets. Notes: Probabilities for treatment choice of mouth film versus tablets among subgroups representing variables that showed significant associations (*p*-value < 0.05) from the extended logistic regression model for the questionnaire study. The solid green line represents the predicted probability of treatment choice, calculated using the mean coefficient estimate from the extended model. The dashed line represents the predicted probability of indifference between the treatment choices. Dissatisfied with current treatment and satisfied with current treatment were constructed by the three lowest scores and the three highest scores for satisfaction with current treatment, ranging 0–10, respectively. Low severity of most recent AAR and high severity of most recent AAR were constructed by the three lowest scores and three highest scores for severity of most recent AAR in the range of 1–11, respectively. Error bars denote standard errors (SE). Abbreviations: AAR: acute allergic reaction.

Figure 2 shows predicted probabilities of treatment choice between characteristics associated with significant differences in the preference for mouth film versus tablets based on treatment choice as indicated by the extended model. For characteristics represented by categorical variables, the figure shows probabilities for participants representing the three highest and the three lowest category values. The figure shows that, although some characteristics were associated with a lower relative probability of choosing the mouth film than others, the overall preference for the mouth film over tablets remained stable. Participants with a shorter history of allergy (<5 years) showed the lowest probability of choosing the mouth film over tablets; the average probability was just above the 0.5 value that marks indifference in terms of preference.

### 3.3. Interim and Ex Post Preferences—Analyses of Clinical Trial Data

#### 3.3.1. Satisfaction with Accessibility

A total of 172 observations were collected for the 41 participants over the 6-month trial period. From the descriptive responses, a preference for the mouth film based on satisfaction with accessibility was reported in 49.4% of the 172 observations, whereas a preference for tablets was reported in 3.5% of the observations. In 47.1% of the observations, no preference for either mouth film or tablets was reported (i.e., indifference).

The regression analysis showed a significant negative association between male sex and satisfaction with accessibility for the mouth film versus tablets, meaning that, although men had an overall preference for the mouth film versus tablets, they had lower relative preference for the mouth film compared with women in terms of satisfaction with accessibility (*p* = 0.023). The detailed regression matrix for the ordinal logistic regression model can be found in the Supplementary Materials (Table S3A).

**Figure 3:**
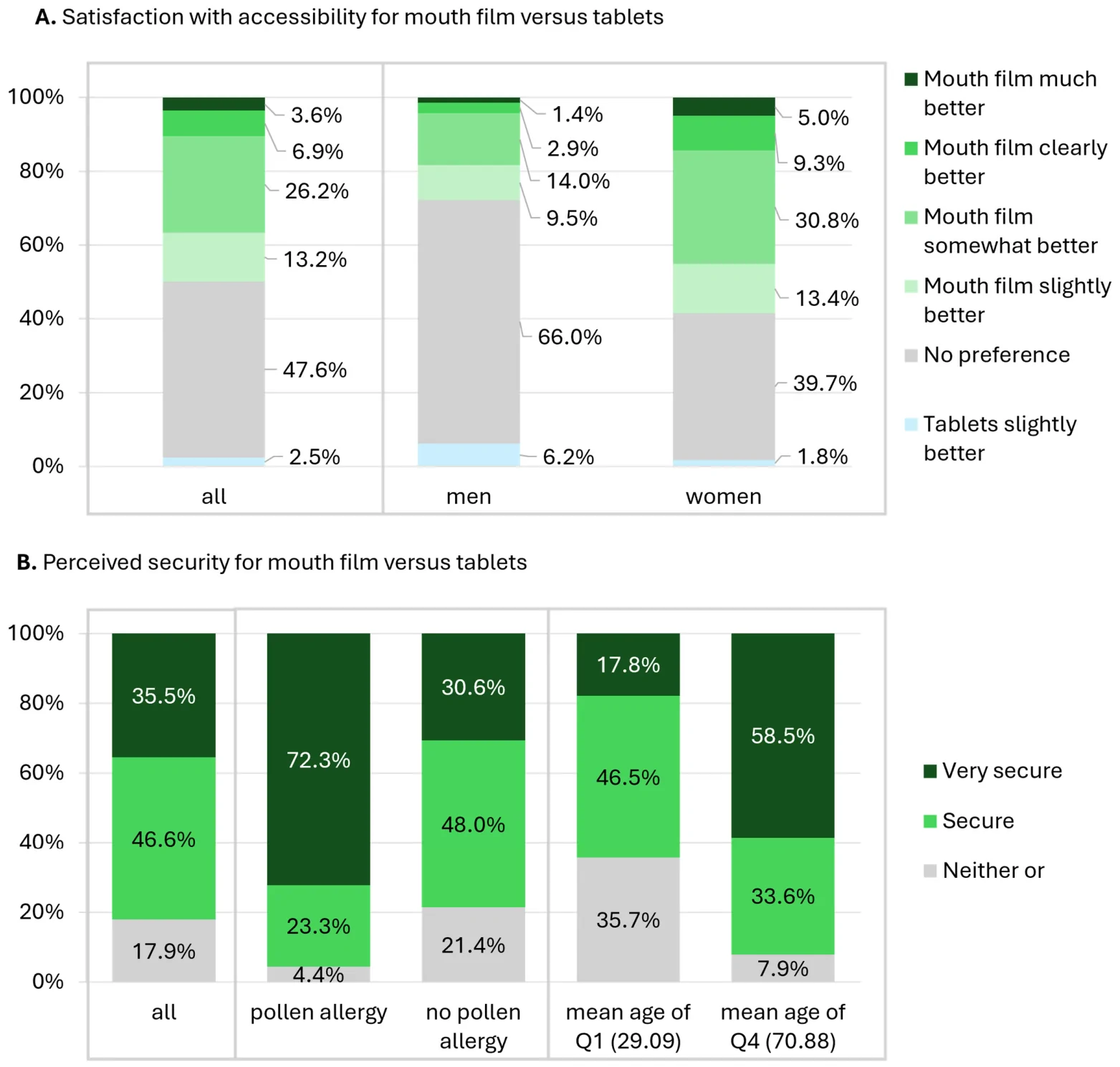
Ratings for preference variables and between characteristics. Notes: Ratings for the preference variables representing satisfaction with accessibility and perceived security for the total sample (172 observations) and between characteristics representing variables that were significantly associated with preference in the ordinal logistic regression analysis of the clinical trial data. Note that in (**B**), responses also included “Not at all secure” and “A little secure”. For detailed calculations, see the Supplementary Materials (Tables S3B and S4B,C).

Figure 3A presents the estimated differences in satisfaction with accessibility, based on the mean values of the independent variables multiplied by the regression coefficients, evaluated for the full sample and stratified by sex. A more detailed example of the calculation is provided in the Supplementary Materials (Table S3B).

#### 3.3.2. Perceived Security

In 79.5% of the clinical trial observations, participants reported feeling secure or very secure with the mouth film versus tablets, whereas indifference was reported for 20.9%. No participant reported that they “did not feel secure at all” or “felt a little secure” with the mouth film versus tablets in any of the observations. Of note was that, among 29 participants who filled in the eDiary at the last follow-up (month 6), 27 (93%) reported that they had received sufficient information to feel safe using the mouth film throughout the trial.

The regression analysis showed a significant positive association between perceived security with mouth film versus tablets and increasing age (*p* = 0.024), meaning that older participants were more likely to express a higher relative preference for the mouth film in terms of perceived security compared with younger participants. In addition, a positive association between perceived security and pollen allergy (*p* = 0.044) indicated that individuals with pollen allergies had a higher relative preference for the mouth film versus tablets in terms of perceived security than those without pollen allergies. The detailed regression matrix for the ordinal logistic regression model can be found in the Supplementary Materials (Table S4A).

Figure 3B presents the estimated differences in perceived security, based on the mean values of the independent variables multiplied by the regression coefficients, evaluated for the full sample and stratified by age and pollen allergy. A more detailed example of the calculation is provided in the Supplementary Materials (Table S4B,C). Note that, because individuals with pollen allergies only were excluded from the analyses (see Section 2), all included participants with pollen allergies had at least one additional concomitant allergy.

### 3.4. Additional Analysis

#### 3.4.1. Stability of Preferences over Time

The linear mixed-effects model demonstrated a significantly higher accessibility rating for the mouth film compared with the tablets (*p* < 0.001). Participants consistently rated the mouth film as more accessible than the tablets throughout the study period. Neither time (month) nor the treatment-by-month interaction was statistically significant, indicating that accessibility ratings remained stable over the six-month follow-up period and that the difference between formulations did not change over time. The results were consistent in both the original and balanced datasets. Detailed model estimates for both analyses are provided in the Supplementary Materials (Table S5).

#### 3.4.2. Interim Versus Ex Post Preferences

Table 2 illustrates how the 10 participants in the ex post subgroup (i.e., participants who experienced one or more events of AAR(s) treated with mouth film) rated their satisfaction with accessibility (rating range −4 to 4) and perceived security (rating range −2 to 2) for mouth film versus tablet over the trial period in relation to the occurrence of AAR events. In total, ratings were available from 42 observations of 60 possible observations (i.e., 18 observations were missing).

Based on satisfaction with accessibility, the mouth film was preferred over tablets (i.e., rating of >0) in 26 observations (62%), and the tablets were preferred (i.e., rating of <0) in 2 observations (5%). In the remaining observations, the participants reported indifference (i.e., rating of 0). Among 32 observations occurring after an AAR event, the mouth film was preferred in 63% of the observations and the tablets in 3%.

Based on perceived security, the mouth film was preferred over the tablets (i.e., rating of >0) in 33 of the observations (79%). In the remaining observations, the participants reported indifference (i.e., rating of 0). Among the 32 observations occurring after an AAR event, the mouth film was preferred in 72% of the observations and the tablets in 0%.

Statistical comparison between mean ratings from observations before and after an AAR event (with imputed values for the missing observations) showed no significant difference in preferences based on satisfaction with accessibility (mean difference: 0.10; 95% CI: −0.68, 0.87; *p* = 0.808) or perceived security (mean difference: −0.24; 95% CI: −0.63; 0.16; *p* = 0.283). It should be noted that these analyses excluded 3 individuals who had no observations before or after an AAR event. Moreover, comparisons between ratings from observations in the same month of an AAR event and those in months without AAR events showed no statistical difference for satisfaction with accessibility (mean difference: 0.18; 95% CI: −0.50; 0.86, *p* = 0.612) or perceived security (mean difference: −0.20; 95% CI: −0.40; 0.00, *p* = 0.085).

## 4. Discussion

Fast-dissolving mouth films may offer several advantages over conventional tablet formulations, including administration without the need for concomitant water intake, a potentially more rapid onset of therapeutic effect, and improved patient adherence [13]. Critical quality and acceptability attributes of mouth films, including end-user acceptability, have recently been reviewed [14,15], and patient preferences when selecting between different film compositions have been investigated [16,17]. However, to our knowledge, patient preferences regarding the choice between mouth films and tablets have not previously been evaluated.

This study used data from two separate studies on preferences for a self-dissolving corticosteroid mouth film versus conventional tablets among individuals with experience of AARs. The two datasets represented preferences based on different levels of information related to outcomes of the mouth film; the questionnaire study evaluated preferences without access to the mouth film, thus representing ex ante preferences. In contrast, the clinical trial assessed preferences among individuals provided with the mouth film and tablets, which they could choose between in case of an AAR. Although only a proportion of the clinical trial participants actually had an AAR and used the mouth film for this purpose during the trial, we were able to assess both interim preferences among patients who had access to treatment without using it, and ex post preferences among patients who had used the treatment and therefore had direct experience.

One key finding of this study was that participants in both studies preferred the mouth film over tablets, regardless of the level of outcome information available. In the questionnaire study, 74% reported that they would choose the mouth film over tablets assuming the same price and effect. In the clinical trial, 49% reported a preference for the mouth film in terms of satisfaction with accessibility compared with 3.5% for the tablets. Also, 80% reported feeling secure or very secure with the mouth film versus tablets. However, we cannot make a direct comparison of preference magnitude between the questionnaire study and the clinical trial due to considerable differences in the outcome measures used to represent preference.

An important difference between the outcomes is that the treatment choice in the questionnaire study represented a forced choice where the participants had to choose between either mouth film or tablets. In contrast, in the clinical trial, the participants rated their satisfaction with the mouth film and tablets and could choose an “indifference” option. Forced choices produce clearer preferences [18], likely increasing the proportion of patients favoring the mouth film as indifferent patients are compelled to choose. Moreover, the clinical trial measured treatment preference using two outcomes, satisfaction with accessibility and perceived security, which capture only specific dimensions of preference. In contrast, treatment choice in the questionnaire study likely reflects a more comprehensive construct by integrating multiple individual factors and therefore represents a more representative measure of overall preference.

Interim and ex post preferences within the clinical trial were assessed by comparing satisfaction with accessibility and perceived security before and after participants gained experience using the mouth film to treat an AAR. Preference for the mouth film persisted following treatment experience, indicating stability of patient-reported outcomes over time. Consistent with this, statistical analyses revealed no significant difference between observations in the months preceding versus following AAR events, nor between observations in the months with and without AARs, indicating that treatment experience did not materially influence preferences. These findings should, however, be interpreted with caution. The sample size for interim versus ex post comparisons was limited; only 10 participants treated an AAR with the mouth film during the trial, and only seven contributed both pre- and post-AAR observations. In addition, the use of imputed values to address missing data may have introduced bias, further limiting the robustness of these results.

Regression analyses showed that, although a preference for the mouth film over tablets was stable independent of those patient characteristics that were included in the regression models, participant characteristics influenced the strength of the preference. In the ex ante situation (i.e., the questionnaire study), there was a stronger preference for the mouth film versus tablets among participants with food allergies, longer allergy duration (>5 years) and those rating the severity of their most recent AAR in the higher range. A plausible explanation for the higher preference in these groups could be that they had more extensive experiences with AARs and acute treatment situations. Individuals who suffer from severe AARs, as is common in food allergies, and those who have a long-term history of AARs have gained more extensive experience of the practical and psychological challenges of managing acute reactions in a real-world situation. These individuals may thus be more aware of, and sensitive to, treatment barriers such as not having the medication or water for administration accessible. Their experiences likely increase the perceived value of dosage forms that are simpler and faster to administer and that can reduce both practical and behavioral barriers to early treatment. This is also consistent with current recommendations stating that corticosteroids, when used, should not delay other essential treatments such as epinephrine [19].

We also found that participants who were satisfied with their current treatment (i.e., betamethasone tablets) and those who had regular healthcare visits due to AARs had a lower, but still positive, preference for the mouth film versus tablets compared with their counterparts. A plausible explanation is that satisfaction with existing therapy reduces the perceived benefit of switching to a new formulation, a phenomenon consistent with status quo bias and loss aversion [20,21]. Similarly, patients with frequent healthcare contacts may feel more protected by established care pathways and therefore place less value on innovations aimed at simplifying self-administration in out-of-hospital settings. In both groups, the perceived incremental benefit of the mouth film may therefore be more limited.

For the clinical trial, the regression analyses were based on all observations collected over the trial period and covered both interim and ex post preferences. To analyze whether user experience of treatment alternatives impacted preferences over time, we conducted a longitudinal regression analysis (mixed-effects repeated-measures model). Although the present study did not directly evaluate learning or device mastery, the longitudinal findings provide some insight into how participants experienced the mouth film over time. First, participants assigned significantly higher ratings of accessibility to the mouth film than to tablets throughout the study period. Second, the treatment difference remained essentially unchanged throughout follow-up, with no evidence that the relative advantage of the mouth film increased or diminished over time. Collectively, these findings suggest that participants established stable perceptions of the mouth film early in the study and maintained these evaluations over the six-month period. Thus, the preferences do not appear to have been influenced by user experience or by the strong familiarization effects observed in some other preference studies [22,23,24]. However, the interpretation of the results in the current study is limited by an unbalanced representation of individuals, with differing numbers of observations across participants. In the context of preferences over time, it should also be mentioned that participants received instructions on how to use the mouth film only at the start of the trial and that no further instructions were provided over the trial period. Nevertheless, at the last follow-up, most participants (27/29) reported that they had received sufficient information to feel safe using the mouth film throughout the trial.

Regression analysis of preferences based on satisfaction with accessibility from the clinical trial showed a significant association between male sex and a lower preference for the mouth film versus tablets compared with female sex. The association may reflect known sex differences in health behavior and treatment preferences [25,26]. Women tend to, for instance, place a greater value on convenience, ease of use, and usability of medication, whereas men may be more accustomed to traditional tablet formulations and focus primarily on perceived efficacy. Differences in experience managing AARs and handling stress may also contribute to this pattern [25,26].

The regression analysis of preferences from the clinical trial based on perceived security showed a higher preference for the mouth film versus tablets among participants with pollen allergy and among older participants. Because participants with pollen allergy only were excluded from the analyses (see Section 2), individuals with pollen allergy who remained in the sample had at least one additional allergy. Therefore, the observed association may reflect a greater preference for the mouth film compared with tablets among individuals with multiple allergies, rather than a specific association with pollen allergy itself. Association with increasing age, similar to longer allergy duration, may indicate longer experience with AARs and acute treatment situations.

When interpreting the results of the clinical trial and the questionnaire study, it is important to consider that the two study populations differed in several clinical characteristics that may have influenced treatment preferences. Participants in the clinical trial were more likely to have epinephrine autoinjector prescriptions, whereas participants in the questionnaire survey generally had a longer history of allergy and a greater number of concomitant allergies. These differences may reflect variation in disease severity, healthcare utilization, and previous experience with allergy management. Such factors could influence how patients value treatment attributes, particularly those related to accessibility, ease of administration, and the confidence or reassurance associated with carrying medication for use during an acute allergic reaction.

The two studies also differed substantially in sample size. The clinical trial only included 44 participants compared with 387 respondents in the questionnaire survey. Consequently, the clinical trial had lower statistical power to detect associations between patient characteristics and treatment preferences, resulting in fewer statistically significant covariates in multivariable analyses. While several patient characteristics were associated with treatment preferences in the questionnaire study, only a limited number reached statistical significance in the clinical trial. This difference should therefore be interpreted cautiously, as it may primarily reflect differences in statistical precision rather than true differences in the underlying relationships between patient characteristics and preferences. Another factor worth considering is that participants in the clinical trial were recruited from a single primary care center located in a university town, which may limit the generalizability of the findings to other healthcare settings. Patients attending this center may differ from patients in other geographical areas with respect to sociodemographic characteristics, educational background, health literacy, and familiarity with healthcare services.

It is also important to note that the regression analyses were limited to variables available in the two datasets. Other demographic or clinical characteristics, such as the duration and frequency of AARs and caregiver availability, may also influence the preference for the mouth film versus tablets.

A key strength of the study was the use of complementary study designs to evaluate distinct dimensions of patient preference. Despite differences in recruitment strategies, sample sizes, and patient characteristics, the pattern of treatment preferences was highly consistent across both studies. In both populations, the mouth film was generally preferred over tablets, supporting the robustness and reproducibility of the primary findings. The larger questionnaire survey afforded enhanced statistical precision and broader population representation, whereas the clinical trial provided longitudinal experience-based insights following real-world use. Together, these complementary approaches strengthen confidence in the findings while underscoring the importance of future evaluation in larger and more diverse patient populations.

## 5. Conclusions

A preference for the mouth film over tablets persisted as the level of outcome information increased and was stable independent of participant characteristics. A particularly strong relative preference for the mouth film was associated with certain characteristics that indicated more experience with acute treatment situations.

## Figures and Tables

**Table 1:** Participant demographics and clinical history.

Study	Questionnaire Study (Ex Ante)	Clinical Trial (Interim + Ex Post)			
Sample Size	*n* = 387	All (*n* = 41)	Interim Subgroup (No AARs Treated with Mouth Film) *n* = 31 ^a^	Ex Post Subgroup (AARs Treated with Mouth Film) *n* = 10	*p*-Value (Interim vs. Ex Post) ^b^
Age, mean (SD)	40.93 (13.70)	49.93 (15.90)	51.90 (16.30)	43.80 (12.87)	
Men, *n* (%)	79 (20.41)	11 (26.83)	7 (22.58)	4 (40.00)	
Women, *n* (%)	308 (79.59)	30 (73.17)	24 (77.42)	6 (60.00)	
BMI, mean (SD)	-	26.24 (4.27)	25.20 (3.48)	29.47 (4.87)	**
Obesity, *n* (%)	-	6 (14.63)	1 (3.23)	5 (50.00)	***
Epinephrine autoinjector prescription, *n* (%)	129 (33.33)	24 (58.54)	19 (61.29)	5 (50.00)	
**Allergy Overview**					
**Last AAR event ^c^**					
Past 6 months ago, *n* (%)	95 (24.55)	12 (29.27)	2 (6.45)	10 (100.00)	***
Between 6 months and 1 year ago, *n* (%)	100 (25.84)	-	-	-	-
More than a year ago, *n* (%)	185 (47.80)	-	-	-	-
Do not know, *n* (%)	7 (1.81)	-	-	-	-
**Allergy Duration (years)**					
Allergy Duration, mean (SD)	-	14.35 (11.85)	14.54 (12.54)	13.76 (9.45)	
Allergy Duration < 2 years, *n* (%)	12 (3.10)	2 (4.88)	2 (6.45)	0 (0.00)	
Allergy Duration 2–5 years, *n* (%)	39 (10.08)	8 (19.51)	5 (16.13)	3 (30.00)	
Allergy Duration > 5 years, *n* (%)	331(85.53)	31 (75.61)	24 (77.42)	7 (70.00)	
Do not know, *n* (%)	5 (1.29)	-	-	-	-
**Number of allergies**					
≥2, *n* (%)	317 (81.91)	9 (21.95)	5 (16.13)	4 (40.00)	
≥3, *n* (%)	184 (47.55)	3 (7.32)	2 (6.45)	1 (10.00)	
**Type of allergy**					
Pollen, *n* (%)	282 (72.87)	5 (12.20)	3 (9.68)	2 (20.00)	
Animal, *n* (%)	222 (57.36)	1 (2.44)	0 (0.00)	1 (10.00)	
Insect sting, *n* (%)	106 (27.39)	13 (31.71)	11 (35.48)	2 (20.00)	
Food, *n* (%)	233 (60.21)	16 (39.02)	13 (41.94)	3 (30.00)	
Other, *n* (%)	116 (29.97)	16 (39.02)	10 (32.26)	6 (60.00)	

Notes: ^a^ Includes individuals without AARs during the trial and two individuals who experienced ARRs which they chose to treat with tablets. ^b^ *p*-values were calculated by comparing the two subgroups in the clinical trial (interim subgroup and ex post subgroup) using a two-proportion z-test for categorical variables and an independent-samples *t*-test for continuous variables (based on mean, SD and sample size); stars indicate statistically significant differences between groups (** = *p* < 0.01, and *** = *p* < 0.001). ^c^ Clinical Trial and Questionnaire Study differ in incidence of question regarding Last AAR event. Abbreviations: AAR: acute allergic reaction; BMI: body mass index; SD: standard deviation.

**Table 2:** Ratings of satisfaction with accessibility and perceived security.

**Satisfaction with Accessibility: Range −4 (Tablets Much Better) to 4 (Mouth Film Much Better)**						
**ID**	**Month 1**	**Month 2**	**Month 3**	**Month 4**	**Month 5**	**Month 6**
1	1 *	2	4	2	2	2
2	3	3	2 *	3	3	3
3	−1	−1	−1	−1 *	0 *	0 *
4	1	2	3 *	2	2	3
5	0 *	0	0	0	0	0 *
6	2	2	2	0 *	0	0
7	0	0 *	0	0	0	0
8	−1	0	0	0	0	1 *
9	3 *	2	4 *	3	3	3
10	0	3	0	3	3 *	0
**Perceived Security: Range −2 (Not Secure at All) to 2 (Very Secure)**						
**ID**	**Month 1**	**Month 2**	**Month 3**	**Month 4**	**Month 5**	**Month 6**
1	0 *	0	1	1	0	0
2	2	2	1 *	1	1	1
3	1	1	1	1 *	1 *	1 *
4	1	1	1 *	2	1	1
5	1 *	1	1	0	0	0 *
6	2	2	2	2 *	2	2
7	0	0 *	0	0	0	0
8	1	1	1	1	1	0 *
9	2 *	2	2 *	2	2	2
10	2	2	2	2	0 *	2

Notes: Ratings of satisfaction with accessibility (interval −4 to 4) and perceived security (interval −2 to 2) for mouth film versus tablets among participants in the ex post subgroup (i.e., individuals who experienced AAR(s) treated with mouth film). Ratings of >0 indicate a preference for the mouth film and <0 indicate a preference for the tablets. * indicate months with AAR event(s) and gray cells indicate month(s) with missing observations where imputed values were used for a balanced dataset in statistical comparisons.

## Data Availability

Data not provided in the main manuscript or in the Supplementary Materials can be provided upon reasonable request.

## Supplementary Materials

The following supporting information can be downloaded at: https://www.mdpi.com/article/10.3390/jmahp14030049/s1. Table S1A: Description of independent variables used in Questionnaire Study analyses; Table S1B: Description of independent variables used in Clinical Trial analyses; Table S2: Regression results from analysis of treatment preferences (mouth film = 1, tablets = 0) from the Questionnaire Study using a logistic regression model for basic model and extended model; Table S3A: Regression results from analysis of satisfaction with accessibility of mouth film versus tablets (−4: tablets much better to 4: mouth film much better) from the Clinical Trial using an ordinal logistic regression model; Table S3B: Calculating Category Probabilities from Cut-Off Values for Males and Females; Table S4A: Regression results from analysis of perceived security of mouth film versus tablets (−2: not secure at all to 2: very secure) from the Clinical Trial using an ordinal logistic regression model; Table S4B: Calculating Category Probabilities from Cut-Off Values for individuals having pollen allergy and individuals not having pollen allergy; Table S4C: Calculating Category Probabilities from Cut-Off Values for mean age of quartile 1 (29.09) and mean age of quartile 4 (70.88); Table S5: Linear mixed-effects model of accessibility rating for the original and balanced sample.
