# Supplementary material for: Treatment Preferences in Acute Allergic Reactions—How Can the Context Impact the Preference for a Corticosteroid Mouth Film Versus Conventional Tablets?
Source: J Mark Access Health Policy. 2026 Aug 11;14(3):49. doi: 10.3390/jmahp14030049 (PMC13511562; doi:10.3390/jmahp14030049)
Supplement: Supplementary file 1 [file jmahp-14-00049-s001.zip › jmahp-4377328-supplementary.pdf]

Karadak et al. Supplementary Material

Supplementary Table S1A. Description of independent variables used in Questionnaire Study analyses

**Variable**	**Description**
Age	Continuous variable measuring each individual's age in years
Sex	Dummy variable taking the value 1 if the individual is a male and 0 if the individual is a female
AAR past 6 months	Dummy variable taking the value 1 if the individual has experienced an acute allergic reaction the past 6 months and 0 otherwise
Epinephrine autoinjector prescription	Dummy variable taking the value 1 if the individual has a prescribed epinephrine autoinjector and 0 otherwise
Allergy duration; more than 5 years	Dummy variable taking the value 1 if the individual has had allergy for more than 5 years and 0 otherwise
3 or more allergies	Dummy variable taking the value 1 if the individual has 3 or more allergies and 0 otherwise
Pollen allergy	Dummy variable taking the value 1 if the individual has pollen allergy and 0 otherwise
Food allergy	Dummy variable taking the value 1 if the individual has food allergy and 0 otherwise
Fur allergy*	Dummy variable taking the value 1 if the individual has fur allergy and 0 otherwise
Insect allergy*	Dummy variable taking the value 1 if the individual has insect allergy and 0 otherwise
Other allergies	Dummy variable taking the value 1 if the individual has other allergies and 0 otherwise
University education*	Dummy variable taking the value 1 if the individual has university education and 0 otherwise
Employment*	Dummy variable taking the value 1 if the individual has employment and 0 otherwise
Household size*	Categorical variable representing household size, including both adults and children (min 1, max 7)
Number of AARs past year*	Categorical variable representing the total number of acute allergic reactions past year (min 0, max 10)
Satisfaction with current treatment*	Categorical variable representing the satisfaction with current treatment based on the question: "How secure do you feel with your current allergy treatment?" (0=very unsecure to 10=very secure)
AAR medication compliance*	Dummy variable based on the question: "Have you ever experienced not having your allergy medication immediately available when you needed it during an acute allergic reaction?" (1=yes, 0=do not know/now)
Swallowing difficulties*	Dummy variable based on the question: "Have you ever experienced difficulty swallowing your allergy medication during an acute allergic reaction?" (1=yes, 0=do not know/now)

Anxiety of AAR*	Categorical variable representing anxiety of acute allergic reaction based on the question: “Are you worried about having an acute allergic reaction?” (0=very worried to 10=not worried at all)
AAR with fear for life*	Dummy variable based on the question: “Have you ever experienced an acute allergic reaction that led you to fear for your life?” (1=yes, 0=do not know/no)
Regular health care check-ups*	Dummy variable based on the question: “Do you attend regular follow-up appointments within the healthcare system for your allergy (at least once per year)?” (1=yes, 0=do not know/no)
Emergency visit(s) due to AAR*	Dummy variable based on the question: “Have you ever required emergency medical care as a result of an acute allergic reaction?” (1=yes, 0=do not know/no)
Severity of most recent AAR*	Categorical variable representing severity of most recent acute allergic reaction based on the question: “How severe was your most recent acute allergic reaction?” (1=very mild to 11=very severe)
Number of tablets for AAR*	Categorical variable representing the number of tablets used for acute allergic reaction based on the categories: 1=fewer than 5 tablets, 2=5-10 tablets, 3=11-15 tablets, 4=16-20 tablets, 5=more than 20 tablets

*Denotes additional independent variables added in the extended model

Supplementary Table S1B. Description of independent variables used in Clinical Trial analyses.

Variable	Description
Age	Continuous variable measuring each individual's age in years
Sex	Dummy variable taking the value 1 if the individual is a male and 0 if the individual is a female
Obesity	Dummy variable taking the value 1 if the individual is obese and 0 otherwise, based on conventional threshold (BMI ≥30)
AAR past month	Dummy variable taking the value 1 if the individual has experienced an acute allergic reaction past month and 0 otherwise
Epinephrine autoinjector prescription	Dummy variable taking the value 1 if the individual has a prescribed epinephrine autoinjector and 0 otherwise
Allergy duration; more than 5 years	Dummy variable taking the value 1 if the individual has had allergy for more than 5 years and 0 otherwise
3 or more allergies	Dummy variable taking the value 1 if the individual has 3 or more allergies and 0 otherwise
Pollen allergy	Dummy variable taking the value 1 if the individual has pollen allergy and 0 otherwise
Food allergy	Dummy variable taking the value 1 if the individual has food allergy and 0 otherwise
Other allergies	Dummy variable taking the value 1 if the individual has other allergies and 0 otherwise

Supplementary Table S2. Regression results from analysis of treatment preferences (mouth film=1, tablets=0) from the Questionnaire Study using a logistic regression model for basic model and extended model

	Basic model		Extended model	
**Variable**	**Coefficient (SD)**	**p-value**	**Coefficient (SD)**	**p-value**
Age	0.01799 (0.0097)	0.064	0.0180 (0.0106)	0.090
Sex†	-0.0843 (0.3067)	0.784	0.0195 (0.3380)	0.954
AAR past 6 months†	0.1880 (0.2986)	0.529	0.5081 (0.3619)	0.160
Epinephrine autoinjector prescription†	0.2150 (0.2751)	0.435	-0.0739 (0.3320)	0.824
Allergy duration; more than 5 years†	1.4956 (0.3676)	**0.000**	1.4422 (0.4065)	**0.000**
3 or more allergies†	0.1781 (0.3386)	0.599	0.3573 (0.5710)	0.532
Pollen allergy†	-0.4711 (0.3490)	0.177	-0.4589 (0.3934)	0.243
Food allergy†	0.8597 (0.2986)	**0.004**	0.8982 (0.4397)	**0.041**
Fur allergy†			-0.3050 (0.4335)	0.482
Insect allergy†			0.2379 (0.4455)	0.593
Other allergies†	0.1037 (0.2939)	0.724	0.0140 (0.4143)	0.973
University education†			0.1708 (0.2973)	0.566
Employed†			0.0206 (0.3084)	0.947
Household size			0.0838 (0.1182)	0.478
Number of AARs past year			-0.0940 (0.0770)	0.222
Satisfied with current treatment			-0.1789 (0.0727)	**0.014**
AAR medication compliance†			0.1001 (0.2891)	0.729
Swallowing difficulties†			0.0063 (0.3420)	0.985
Anxiety of AAR			-0.0274 (0.0608)	0.652
AAR with fear for life†			-0.0493 (0.3111)	0.874
Regular health care check-ups†			-1.0342 (0.2902)	**0.000**
Emergency visit(s) due to AAR†			-0.1947 (0.3121)	0.533
Severity of most recent AAR			0.1640 (0.0721)	**0.021**
Number of tablets for AAR			0.2722 (0.1541)	0.077
Constant	-1.2565 (0.5252)	**0.017**	-1.1247 (1.0442)	0.281
Number of observations	387		387	
Wald chi2	44.91		81.07	
Prob > Chi2	**0.0000**		**0.0000**	
Pseudo R2	0.1006		0.1816	
Log likelihood	-200.74799		-182.66662	

Notes: In a logistic regression model, the outcome is binary, choosing mouth film versus tablets, and independent variables affect the likelihood of choosing the mouth film relative to tablets. Coefficients represent the log-odds of choosing the mouth film relative to the reference group. Positive coefficients indicate a greater likelihood of choosing mouth film compared with the reference group, while negative coefficients indicate a lower likelihood compared with the reference group. These coefficients reflect relative differences within the sample, not absolute probabilities of choosing the mouth film.

Abbreviations; AAR: acute allergic reactions; SD: standard deviation

†Denoting dummy variables. The variable equals 1 if the condition is met and 0 otherwise (e.g., pollen allergy = 1, no pollen allergy = 0).

Supplementary Table S3A. Regression results from analysis of satisfaction with accessibility of mouth film versus tablets (-4: tablets much better to 4: mouth film much better) from the Clinical Trial using an ordinal logistic regression model

Dependent variable: Satisfaction with accessibility		
**Variable**	**Coefficient (SD)**	**p-value**
Age	-0.0150 (0.1666)	0.368
Sex†	-1.3003 (0.5728)	**0.023**
Obesity†	0.1076 (1.0078)	0.915
AAR past month†	-0.4102 (0.7867)	0.602
Epinephrine autoinjector prescription†	0.4489 (0.5404)	0.406
Allergy duration; more than 5 years†	0.5395 (0.8155)	0.508
3 or more allergies†	-1.2870 (1.0269)	0.210
Pollen allergy†	1.2820 (0.8681)	0.140
Food allergy†	0.0691(0.6311)	0.913
Other allergies†	1.5460 (0.8306)	0.063
Cut 1: Tablets slightly better vs No preference	-3.51086 (1.4494)	
Cut 2: No preference vs Mouth film slightly better	0.1645 (1.3360)	
Cut 3: Mouth film slightly better vs Mouth film somewhat better	0.7062 (1.3175)	
Cut 4: Mouth film somewhat better vs Mouth film better	2.3019 (1.3945)	
Cut 5: Mouth film better vs Mouth film much better	3.4650 (1.3441)	
Number of observations	172	
Wald chi2	31.26	
Prob > Chi2	0.0005	
Pseudo R2	0.0738	
Log pseudolikelihood	-227.24174	

Notes: In an ordinal logistic model, the outcome is categorical, from -4: tablets much better to 4: mouth film much better, and independent variables affect the likelihood of being in higher versus lower categories of the outcome. Coefficients represent the log-odds of choosing the mouth film relative to the reference group. Positive coefficients indicate a greater likelihood of choosing mouth film compared with the reference group, while negative coefficients indicate a lower likelihood compared with the reference group. These coefficients reflect relative differences within the sample, not absolute probabilities of choosing the mouth film. Cut-offs define the points that divide the unobserved underlying scale into the observed ordinal categories, and explanatory variables affect the likelihood of falling above or below these cut-offs. In theory, there are seven possible cut-offs for the eight categories. The data only contains six observed categories, the model estimates five cut-offs.

Abbreviations; AAR: acute allergic reactions; SD: standard deviation

†Denoting dummy variables. The variable equals 1 if the condition is met and 0 otherwise (e.g., pollen allergy = 1, no pollen allergy = 0).

Supplementary Table S3B. Calculating Category Probabilities from Cut-Off Values for Males and Females

Calculation Example 1.1
In this example, we calculate the predicted probability associated with the significant variable sex in relation to satisfaction with the accessibility of mouth film compared with tablets (Table S3A). To do this, all other covariates in the regression equation are set to their sample averages (e.g., mean age or the sample fraction for binary variables), while sex is set to male. This yields the predicted probability for males in the sample. The calculation is repeated with sex set to female as well as the overall sample using the sample fraction of males, yielding the corresponding predicted probability for females and the baseline probabilities, respectively.<br><br>*Setting the regression covariates to their sample averages gives the following values: Age = mean age (49.93), Sex = 1 (male), Obesity = fraction of the sample (14.63%), AAR past 6 months = fraction of the sample (29.27%), epinephrine autoinjector prescription = fraction of the sample (58.54%), Allergy duration: more than 5 years = fraction of the sample (75.61%), 3 or more allergies = fraction of the sample (7.32%), Pollen allergy = fraction of the sample (12.20%), Food allergy = fraction of the sample (39.02%), and Other allergies = fraction of the sample (39.02%).*<br><br>*Substituting these values into the regression equation gives the linear predictor:*<br>*η = (-0.0150)(49.93) + (-1.3003)(1) + 0.1076(0.1463) + (-0.4102)(0.2927) + 0.4489(0.5854) + 0.5395(0.7561) + (-1.2870)(0.0732) + 1.2820(0.1220) + 0.0691(0.3902) + 1.5460(0.3902)*<br>*η = −0.7906*<br><br>*The cumulative probability for each response category is calculated using the logistic function:*<br>$$P\ (Y\ \leq j) = logistic\big(cut_j - \eta\big) = \frac{1}{1+e^{-(cut_j - \eta)}}$$<br>*P≤1 = logistic(−3.51086 – (−0.7906)) = logistic(−2.72026) ≈ 0.062*<br>*P≤2 = logistic(0.1645 − (−0.7906)) = logistic(0.9551) ≈ 0.722*<br>*P≤3 = logistic(0.7062 − (−0.7906)) = logistic(1.4968) ≈ 0.817*<br>*P≤4 = logistic(2.3019 − (−0.7906)) = logistic(3.0925) ≈ 0.957*<br>*P≤5 = logistic(3.4650 − (−0.7906)) = logistic(4.2556) ≈ 0.986*<br><br>*The probability for each category is obtained by taking the difference between consecutive cumulative probabilities (1=tablets slightly better, 0=indifferent, 1=mouth film slightly better, 2=mouth film somewhat better, 3=mouth film clearly better, 4=mouth film much better). The five cut-offs in the model come from the actual responses observed:*<br>*P(Y=Tablets slightly better) = 0.062*<br>*P(Y=Indifferent) = 0.722 − 0.062 = 0.660*<br>*P(Y=Mouth film slightly better) = 0.817 − 0.722 = 0.095*<br>*P(Y=Mouth film somewhat better) = 0.957 − 0.817 = 0.140*<br>*P(Y=Mouth film clearly better) = 0.986 − 0.957 = 0.029*<br>*P(Y=Mouth film much better) = 1 − 0.986 = 0.014*<br>*0.062 + 0.660 + 0.095 + 0.140 + 0.029 + 0.014 = 1.00*

These predicted probabilities are presented in Figure 3A to illustrate the differences in satisfaction with accessibility between males and females implied by the regression results.

Supplementary Table S4A. Regression results from analysis of perceived security of mouth film versus tablets (-2: not secure at all to 2: very secure) from the Clinical Trial using an ordinal logistic regression model

Dependent variable: Perceived security		
**Variable**	**Coefficient (SD)**	**p-value**
Age	0.0448 (0.0198)	**0.024**
Sex†	-0.5345 (0.8373)	0.523
Obesity†	-0.3441 (0.6217)	0.580
AAR past month†	-0.1869 (0.5263)	0.722
Epinephrine autoinjector prescription†	0.1126 (0.6764)	0.868
Allergy duration; more than 5 years†	-0.0999 (0.9480)	0.916
3 or more allergies†	-0.2905 (1.3179)	0.826
Pollen allergy†	1.7747 (0.8828)	**0.044**
Food allergy†	-0.1898 (0.6584)	0.773
Other allergies†	-0.2326 (0.7718)	0.763
Cut 1: Neither nor vs Secure	0.4883 (1.5168)	
Cut 2: Secure vs Very secure	2.6087 (1.5628)	
Number of observations	172	
Wald chi2	22.20	
Prob > Chi2	0.0141	
Pseudo R2	0.1125	
Log pseudolikelihood	-161.95909	

Notes: In an ordinal logistic model, the outcome is categorical, from -2: not secure at all to 2: very secure, and independent variables affect the likelihood of being in higher versus lower categories of the outcome. Coefficients represent the log-odds of choosing the mouth film relative to the reference group. Positive coefficients indicate a greater likelihood of choosing mouth film compared with the reference group, while negative coefficients indicate a lower likelihood compared with the reference group. These coefficients reflect relative differences within the sample, not absolute probabilities of choosing the mouth film. Cut-offs define the points that divide the unobserved underlying scale into the observed ordinal categories, and explanatory variables affect the likelihood of falling above or below these cut-offs. In theory, there are three possible cut-offs for the four categories. The data only contains three observed categories, the model estimates two cut-offs.

Abbreviations; AAR: acute allergic reactions; SD: standard deviation

†Denoting dummy variables. The variable equals 1 if the condition is met and 0 otherwise (e.g., pollen allergy = 1, no pollen allergy = 0).

Supplementary Table S4B. Calculating Category Probabilities from Cut-Off Values for individuals having pollen allergy and individuals not having pollen allergy

Calculation Example 2.1

In this example, we calculate the predicted probability associated with the significant variable pollen allergy in relation to perceived security for mouth film compared with tablets (Table S4A). To do this, all other covariates in the regression equation are set to their sample averages (e.g., mean age or the sample fraction for binary variables), while pollen allergy is set to 1, indicating having at least pollen allergy (individuals with only pollen allergy were excluded, as described elsewhere). This yields the predicted probability for individuals with pollen allergy. The calculation is then repeated with pollen allergy set to 0, indicating individuals not having pollen allergy as well as the overall sample using the sample fraction of individuals with pollen allergy, yielding the corresponding predicted probability for individuals not having pollen allergy and the baseline probabilities, respectively.

Setting the regression covariates to their sample averages gives the following values: Age = mean age (49.93), Sex = fraction of males of the sample (26.83), Obesity = fraction of the sample (14.63%), AAR past 6 months = fraction of the sample (29.27%), epinephrine autoinjector prescription = fraction of the sample (58.54%), Allergy duration; more than 5 years = fraction of the sample (75.61%), 3 or more allergies = fraction of the sample (7.32%), Pollen allergy = 1, Food allergy = fraction of the sample (39.02%), and Other allergies = fraction of the sample (39.02%).

Substituting these values into the regression equation gives the linear predictor:
η = (0.0448)(49.93) + (-0.5345)(0.2683) + (-0.3441)(0.1463) + (-0.1869)(0.2927) + 0.1126(0.5854) + (-0.0999)(0.7561) + (-0.2905)(0.0732) + (1.7747)(1) + (-0.1898)(0.3902) + (-0.2326)(0.3902)
η = 3.567

The cumulative probability for each response category is calculated using the logistic function:

$$P\ (Y\ \leq j) = logistic\left(cut_j - \eta\right) = \frac{1}{1 + e^{-(cut_j - \eta)}}$$

P≤1 = logistic(0.4883 – 3.567) = logistic(−3.0787) ≈ 0.044
P≤2 = logistic(2.6087 − 3.567) = logistic(−0.9583) ≈ 0.277

The probability for each category is then obtained by taking the difference between consecutive cumulative probabilities (0=neither nor, 1=secure, 2=very secure). The two cut-offs in the model come from the actual responses observed:
P(Y=Neither nor) = 0.044
P(Y=Secure) = 0.277− 0.044 = 0.233
P(Y=Very secure) = 1 − 0.277 = 0.723
0.044 + 0.233 + 0.723 = 1.00

These predicted probabilities are presented in Figure 3B to illustrate how perceived security differs between individuals with and without pollen allergy.

Supplementary Table S4C. Calculating Category Probabilities from Cut-Off Values for mean age of quartile 1 (29.09) and mean age of quartile 4 (70.88)

Calculation Example 2.2
In this example, we calculate the predicted probability associated with the significant variable age in relation to perceived security for mouth film compared with tablets (Table S4A). To do this, all other covariates in the regression equation are set to their sample averages (e.g., the sample fraction for binary variables), while age is set to the mean age of the first quartile (Q1). This yields the predicted probability for individuals with the mean age of Q1. The calculation is then repeated with age set to the mean age of the fourth quartile (Q4) as well as the overall sample using the sample mean age of the full sample, yielding the corresponding predicted probabilities for individuals who differ only in age while all other characteristics are held constant at their sample averages.<br><br>*Setting the regression covariates to their sample averages gives the following values: Age = mean age in Q1 (29.09), Sex = fraction of males of the sample (26.83), Obesity = fraction of the sample (14.63%), AAR past 6 months = fraction of the sample (29.27%), epinephrine autoinjector prescription = fraction of the sample (58.54%), Allergy duration; more than 5 years = fraction of the sample (75.61%), 3 or more allergies = fraction of the sample (7.32%), Pollen allergy = (12.20%), Food allergy = fraction of the sample (39.02%), and Other allergies = fraction of the sample (39.02%).*<br><br>*Substituting these values into the regression equation gives the linear predictor:*<br>*η = (0.0448)(29.09) + (-0.5345)(0.2683) + (-0.3441)(0.1463) + (-0.1869)(0.2927) + 0.1126(0.5854) + (-0.0999)(0.7561) + (-0.2905)(0.0732) + (1.7747)(0.1220) + (-0.1898)(0.3902) + (-0.2326)(0.3902)*<br>*η = 1.075*<br><br>*The cumulative probability for each response category is calculated using the logistic function:*<br>$$P\ (Y\ \leq j) = logistic\left(cut_j -\ \eta\right) = \frac{1}{1+e^{-(cut_j - \eta)}}$$<br>*P≤1 = logistic(0.4883 – 1.075) = logistic(−0.5867) ≈ 0.357*<br>*P≤2 = logistic(2.6087 – 1.075) = logistic(1.5337) ≈ 0.822*<br><br>*The probability for each category is then obtained by taking the difference between consecutive cumulative probabilities (0=neither nor, 1=secure, 2=very secure). The two cut-offs in the model come from the actual responses observed:*<br>*P(Y=Neither nor) = 0.357*<br>*P(Y=Secure) = 0.822 − 0.357 = 0.465*<br>*P(Y=Very secure) = 1 − 0.822 = 0.178*<br>*0.357 + 0.465 + 0.178 = 1.00*<br><br>*These predicted probabilities are presented in Figure 3B to illustrate how perceived security differs between individuals with ages corresponding to the mean age of the first and fourth quartiles in the sample.*

Supplementary Table S5. Linear mixed-effects model of accessibility rating for the original and balanced sample

	Original sample		Balanced sample	
**Variable**	**Coefficient (SD)**	**p-value**	**Coefficient (SD)**	**p-value**
Treatment	1.1480 (0.1877)	**0.000**	1.0293 (0.1528)	**0.000**
Month	0.0349 (0.0362)	0.334	0.4808 (0.0278)	0.083
Treatment x Month	-0.0340 (0.0502)	0.498	-0.0432 (0.0392)	0.271
Constant	2.5656 (0.1525)	**0.000**	2.6163 (0.1326)	**0.000**
Number of observations	344		492	
Number of groups	41		41	
Observations per group, mean (min; max)	8.4 (2; 12)		12.0 (12; 12)	
Wald chi2 (3)	147.37		174.68	
Prob > Chi2	0.0000		0.0000	
Log pseudolikelihood	-434.30242		-589.74229	

Notes: A linear mixed-effects model with a participant-specific random intercept was fitted. Fixed effects included treatment formulation (tablet as the reference category), month (modeled as a continuous variable), and the treatment-by-month interaction. Regression coefficients represent the estimated difference in accessibility rating relative to the tablets or, for month, the estimated average change in rating per month. The original sample included only observed participant responses (no imputation). The balanced sample included imputed values using the Last Observation Carried Forward (LOCF) and Next Observation Carried Backward (NOCB) methods.

Abbreviations; SD: standard deviation
